# Mass Spectrometry-Based Targeted Serum Monomethylated Ribonucleosides Profiling for Early Detection of Breast Cancer

**DOI:** 10.3389/fmolb.2021.741603

**Published:** 2021-08-26

**Authors:** Zhihao Fang, Yiqiu Hu, Jiani Chen, Kailun Xu, Kailai Wang, Shu Zheng, Cheng Guo

**Affiliations:** ^1^ Cancer Institute (Key Laboratory of Cancer Prevention and Intervention, China National Ministry of Education), The Second Affiliated Hospital, Zhejiang University School of Medicine, Hangzhou, China; ^2^ Cancer Center, Zhejiang University, Hangzhou, China

**Keywords:** HILIC-MS/MS, RNA modification, monomethylated ribonucleosides, serum, breast cancer

## Abstract

RNA methylation plays a significant regulatory role in various of physiological activities and it has gradually become a hotspot of epigenetics in the past decade. 2′-O-methyladenosine (Am), 2′-O-methylguanosine (Gm), 2′-O-methylcytidine (Cm), 2′-O-methyluridine (Um), *N* ^6^-methyladenosine (m^6^A), *N* ^1^-methylguanosine (m^1^G), 5-methylcytidine (m^5^C), and 5-methyluridine (m^5^U) are representative 2′-O-methylation and base-methylation modified epigenetic marks of RNA. Abnormal levels of these ribonucleosides were found to be related to various diseases including cancer. Serum is an important source of biofluid for the discovery of biomarkers, and novel tumor biomarkers can be explored by measuring these ribonucleoside modifications in human serum. Herein, we developed and applied a hydrophilic interaction liquid chromatography tandem mass spectrometry (HILIC-MS/MS) method to determine the content of monomethylated ribonucleosides in human serum. The developed method enabled sensitive and accurate determination of these monomethylated ribonucleosides. By applying this robust method, we demonstrated the presence of Gm and Um in human serum for the first time, and we successfully quantified m^6^A, Gm, m^1^G, Cm, Um and m^5^U in serum samples collected from 61 patients with breast cancer and 69 healthy controls. We discovered that the levels of Gm, m^1^G, Cm, Um and m^5^U in serum were all significantly decreased in breast cancer patients whereas m^6^A was increased. We performed receiver operating characteristic (ROC) curve analysis, and obtained highest area under curve (AUC) value when combining these six monomethylated ribonucleosides together. These results suggest that m^6^A, Gm, m^1^G, Cm, Um and m^5^U might have great potential to be novel biomarkers for detection of breast cancer in the early stage. In addition, this study may stimulate future investigations about the regulatory roles of monomethylated ribonucleosides on the initiation and development of breast cancer.

## Introduction

Post-transcriptional modifications of RNA has great research prospects and RNA epigenetics/epitranscriptomics has been proposed (He, 2010). More than 170 different modifications of RNA have been identified in recent year and over half of them are RNA methylation modifications which are associated with the regulation of RNA functions (Jonkhout et al., 2017; Ontiveros et al., 2019). Due to the functional identification of important regulatory proteins such as methyltransferase METTL3/METTL14/WTAP complex (Liu et al., 2014) and demethylase FTO (Jia et al., 2011), RNA methylation modifications have attracted great attention and accumulating evidences have been obtained to confirm RNA methylation modifications as a novel layer of epigenetic alteration. Through their unique regulatory proteins, RNA methylation modifications play critical roles in various cellular functions, such as RNA splicing (Sun et al., 2020), stability (Zhang et al., 2019a; Chen et al., 2019; Shen et al., 2020), degradation (Ni et al., 2019; Yang et al., 2019; Chen et al., 2020b) and translation (Weng et al., 2018; Schumann et al., 2020; Song et al., 2020). Moreover, it has been revealed that RNA methylation is closely associated with the occurrence and development of human cancers.

As we known, the ribonucleoside compositions of RNA contain adenosine, guanosine, cytidine and uridine. The methylation usually occurs at nitrogen or carbon atom in the nucleobase part of the molecule to form m^6^A, m^1^G, m^5^C, m^5^U and so on. When the hydrogen on the 2′-hydroxyl (-OH) of the ribose moiety is replaced by a methyl group (-CH_3_), it will form 2′-O-methylation ribonucleosides (Nm) including Am, Gm, Cm and Um. M^6^A is the most predominant modification in mRNA, and the aberrant level of m^6^A modification has great connection with the tumorigenesis and development (Ma et al., 2019; Xu et al., 2020; Chen et al., 2021; Fang et al., 2021). In the recent study of our group, it was found that the significantly elevated m^6^A in human serum increased the risk of colorectal cancer and gastric cancer (Hu et al., 2021). It was reported that the dynamic level of m^1^G in human serum could help early detection of breast cancer (Rashed et al., 2020) and colorectal cancer (Zhu et al., 2015). M^5^C is another most abundant RNA modification which has been discovered to be a potential biomarker of various cancers (Guo et al., 2018a; Zhang et al., 2019b; Feng et al., 2020). In addition, a significant downregulation m^5^U in human serum can indicate the presence of prostate cancer (Buzatto et al., 2017). For Nm, they have also been revealed to participate in the pathogenesis of various cancers and play crucial roles (Hua et al., 2018; Zhu et al., 2019; Wu et al., 2020). Recently, Li et al. reported that the level of Cm in serum is related to the reduced risk of developing esophageal squamous cell carcinoma (Li et al., 2021). Therefore, these monomethylated ribonucleosides have significant potential to be used as indicators for early detection of cancers.

Breast cancer is the most prevalent tumor with the highest mortality among women worldwide (Sung et al., 2021). Generally, the prognosis and the survival rate of breast cancer are better in the early stages, but poorer in advanced stages even after receiving the surgery and adjuvant treatment (DeSantis et al., 2016). Therefore, it is important for early detection of breast cancer. Currently, ultrasonography and mammography, together with biopsy, are used for routine screening and staging. Limitations of these inspection methods including exposure to radiation, traumatic operation and often producing inaccurate results. Besides, breast tumors must be at least a few millimeters in size to be detected. Serum biomarkers, such as carbohydrate antigen-153 (CA153) and carcinoembryonic antigen (CEA), showed low sensitivity and/or low specificity and responded late to tumor formation and recurrence (Nagrath et al., 2011). From these points of view, it is necessary to hunt for robust biomarkers for early-stage of breast cancer to elongate the survival time and reduce the suffering of patients.

Serum is easy to obtain in the clinic and contains a large number of biomolecules, so it can be used as the body fluid of choice for the discovery of biomarkers. In the past decades, a variety of analytical methods have been utilized for analyzing modified ribonucleosides (Jiang and Ma, 2009; Wei et al., 2010; Beale et al., 2018; Pero-Gascon et al., 2018). Reversed-phase liquid chromatography (RPLC) or hydrophilic interaction liquid chromatography (HILIC) coupled with tandem mass spectrometry is more favored for biomarker discovery due to its great advantages in selectivity, sensitivity, accuracy and high throughput (Zhu et al., 2015; Guo et al., 2018b; Chen et al., 2020a; Guo et al., 2020; Su et al., 2021), compared with other analytical techniques. In our study, a fast, sensitive, simple and reliable HILIC-MS/MS method for qualitative and quantitative detection of monomethylated ribonucleosides in human serum was established. We revealed the presence of Gm and Um in human serum for the first time and quantified m^6^A, Gm, m^1^G, Cm, Um and m^5^U in serum from breast cancer patients and healthy controls. By analyzing these results, we demonstrated the differences of these modifications between breast cancer patients and healthy volunteers, and evaluate the potential of these monomethylated ribonucleosides as biomarkers for early detection of breast cancer.

## Materials and Methods

### Chemicals and Reagents

Chromatographic grade acetonitrile was bought from Merck KGaA (Darmstadt, Germany). Methanol of HPLC grade was purchased from J.T.Baker (Radnor, PA, United States). Formic acid (HCOOH) was bought from Fluka (Muskegon, United States). Ammonium formate, malic acid and 5-methylcytidine (m^5^C) were bought from Sigma-Aldrich (St Louis, MO, United States). 2′-O-methyladenosine (Am), *N* ^6^-methyladenosine (m^6^A), 2′-O-methylguanosine (Gm), 1-methylguanosine (m^1^G), 2′-O-methylcytidine (Cm), 2′-O-methyluridine (Um), 5-methyluridine (m^5^U) and isotopically labeled standards [D_3_]m^6^A, [D_3_]Um and [^13^C_5_]m^5^U were obtained from Toronto Research Chemicals (Toronto, Canada). [^13^C^15^N_2_]Gm, [^13^C^15^N_2_]m^1^G and [^13^C_5_]Cm were synthesized according to literature (Fu et al., 2015). Water was purified by a Milli-Q water purification device (Millipore, Milford, MA, United States).

### Instrumentation

Acquity UPLC system (Waters, Milford, MA, United States) achieved by Empower Pro 6.0 software was applied for analysis. A Waters Acquity BEH Amide column (100 mm × 2.1 mm, 1.7 μm) was applied for chromatographic separation. 4000 QTRAP mass spectrometer (AB SCIEX, Foster City, CA, United States) was applied for MS detection. The mass spectrometer was equipped with electrospray ionization (ESI) positive ion mode. Multiple-reaction monitoring (MRM) was chosen to acquire data. Data acquisition and processing were controlled by Analyst 1.6.3 software.

### Sample Collection

The Ethics Committee of Medical Research of the Second Affiliated Hospital, Zhejiang University School of Medicine (SAHZU) approved our study. A total of 69 healthy volunteers (mean age of 43.9 ± 11.1 years, range from 30 to 70 years) and 61 patients with breast cancer (mean age of 52.2 ± 11.9 years, range from 29 to 80 years) were recruited from SAHZU. All breast cancer patients had a diagnosis report with a pathological stage of stage I or stage II at SAHZU between June 2020 and December 2020. The exclusion criteria were as follows: 1) Co-suffering other malignant tumors. 2) Having received any type of treatment for tumors. 3) Suffering from metabolic diseases, kidney diseases or liver diseases. 4) Taking any drugs for a long time. All participating volunteers agreed the informed consent in advance. Then, the serum samples were collected in the early morning and reserved at −80°C.

### Sample Preparation

At first, 10 μl of isotope-labeled internal standards (IS) mixed solution was added into 100 μl serum samples which were naturally thawed in ice. And then 330 μl pre-refrigerated acetonitrile/methanol of 2:1 (v/v) was added. After vortexed for 60 s, let it stand at −20°C for 2 h and centrifuged at 13,000 rpm, 4°C for 15 min orderly, 352 μl of the supernatant were taken out and then drain under vacuum. Then, 80 μl acetonitrile/water of 9:1 (v/v) was used to redissolve the dried samples. After vortexed for 10 s, ultrasonicated for 15 s and centrifuged at 13,000 rpm for 15 min at 4°C, 70 μl of the supernatant fraction were sucked into the sample bottle for subsequent HILIC-MS/MS detection.

### HILIC-MS/MS Analysis

The mobile phase was (A) H_2_O containing 10 mM ammonium formate, 0.2% formic acid and 0.06 mM malic acid, and (B) acetonitrile containing 2 mM ammonium formate, 0.2% formic acid and 0.06 mM malic acid. The eight analytes were perfectly separated at a flow rate of 0.4 ml/min by the optimized LC gradient program as follows: 0 min, 94% B; 4 min, 94% B; 6.1 min, 75% B; 6.5 min, 94% B; 8 min, 94% B. The BEH Amide column was set at 40°C and the samples temperature was maintained at 4°C. 5 μl of sample was injected each time and each sample was measured twice. To minimize the interference of the mass spectrometer, a switching valve was used and the eluents from the column were introduced into the ion source during 1.0–6.5 min.

The ion spray voltage was kept at 5.5 kV and the ion source temperature (TEM) was maintained at 550°C. Ion source gas 1 (GS1), ion source gas 2 (GS2) and curtain gas (CUR) were all set at 45 psi. The ion transitions of these eight ribonucleosides and corresponding isotope labeled internal standard (IS) were shown in Supplementary Table S1. The optimized MRM parameters of them including declustering potential (DP), entrance potential (EP), collision energy (CE) and collision cell exit potential (CXP) were also listed in Supplementary Table S1.

### Method Validation

The standard working solutions of m^6^A, Gm, m^1^G, Cm, Um and m^5^U at different concentrations (1, 2.5, 5, 10, 25, 50, 100, 250, 500 nM), which were mixed with IS solution (final concentration: [D_3_]m^6^A (5 nM), [^13^C^15^N_2_]Gm (10 nM), [^13^C^15^N_2_]m^1^G (20 nM), [^13^C_5_]Cm (30 nM), [D_3_]Um (20 nM) and [^13^C_5_]m^5^U (100 nM)), were made and analyzed. The calibration curves could describe as y = ax + b, where y represents the peak area ratio of the analyte to the corresponding IS and x denotes the concentration of the analyte. The limit of detection (LOD) and limit of quantification (LOQ) of each ribonucleoside were obtained by analyzing standard solutions with a signal-to-noise ratio of three and ten, respectively.

For the purpose of evaluating intra-day and inter-day precision, the quality control (QC) samples at three different levels of m^6^A (2.5, 5, 50 nM), Gm (5, 10, 50 nM), m^1^G (5, 20, 100 nM), Cm (5, 30, 150 nM), Um (5, 20, 100 nM) and m^5^U (20, 100, 300 nM) were prepared in triplicate and were measured on the same day and three consecutive days, respectively. The accuracy was described as the ratio of measured value to the theoretical concentration.

For the purpose of evaluating the recovery of extraction, the serum samples were added with three different levels of m^6^A (2.5, 5, 30 nM), Gm (2.5, 10, 50 nM), m^1^G (5, 20, 60 nM), Cm (6, 30, 150 nM), Um (5, 20, 60 nM) and m^5^U (50, 150, 300 nM). After 10 μl of IS solution (same as described above) was added, the serum samples were treated and analyzed as mentioned above. The recovery (R) of each analyte was calculated by (concentration in added serum sample–concentration in original serum sample)/added concentration × 100%.

The matrix effect was estimated by using a slope comparison method. By adding different concentrations (1, 2.5, 5, 10, 25, 50, 100, 250, 500 nM) standard solution and IS to the serum extracts, the calibration curve was obtained. The ratio value of its slope to the slope of the standard solution calibration curve was matrix effect.

### Statistical Analysis

Statistical analyses were achieved through SPSS 24.0 software (IBM, Armonk, NY, United States). The concentration differences of serum monomethylated modifications between healthy volunteers and breast cancer patients were accessed by Two-tailed Student’s t-test, where *p* value less than 0.05 was considered meaningful. The area under the curve (AUC) was acquired by receiver operating characteristic (ROC) curve analysis, and the optimal cut-off value of the methylation modification in serum for the diagnosis of breast cancer was determined by the Youden index (Youden index = sensitivity + specificity −1). Receiver operating characteristic (ROC) analysis was performed to evaluate the ability of monomethylated modifications to distinguish cancer patients from healthy controls.

## Results and Discussion

### Optimization of Chromatographic Conditions and Mass Spectrometry Parameters

**FIGURE 1:**
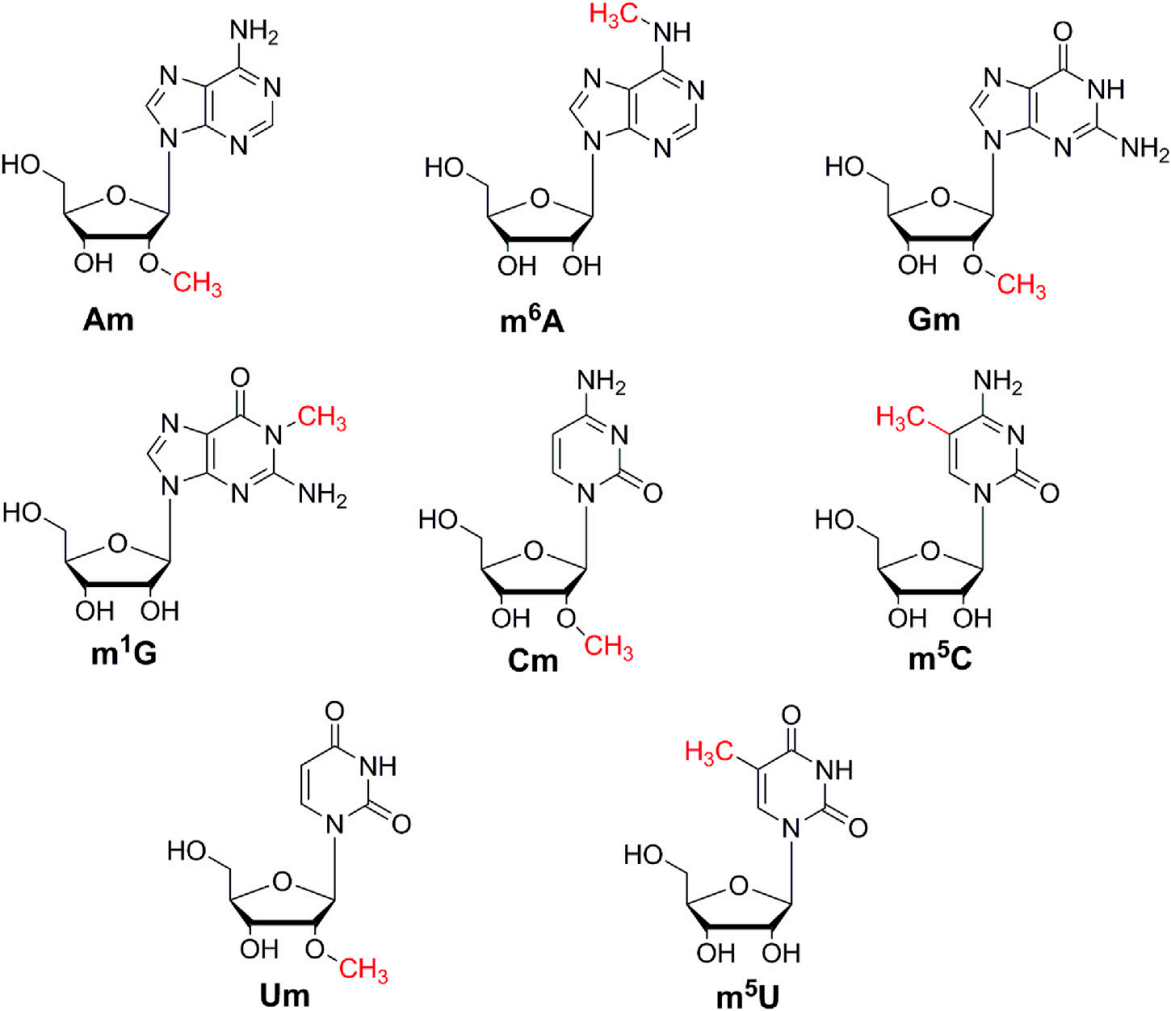
The chemical structures of Am, m^6^A, Gm, m^1^G, Cm, m^5^C, Um, m^5^U.

**FIGURE 2:**
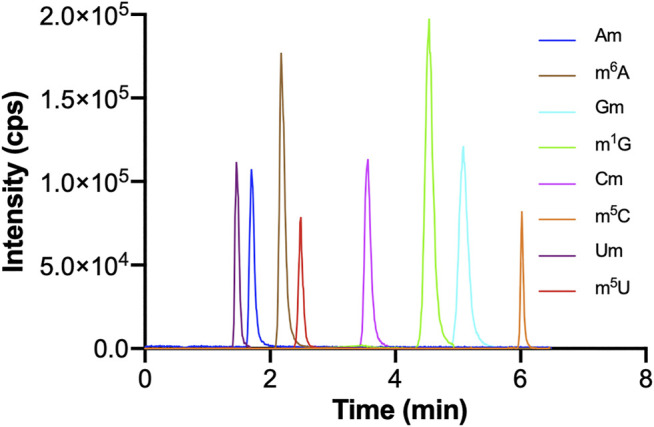
The MRM chromatograms of Am, m^6^A, Gm, m^1^G, Cm, m^5^C, Um, m^5^U standards. The concentrations of Um and m^5^U were 1,000 nM, and the concentrations of other nucleosides standards were 100 nM. The injection volume was 5.0 μl.

The optimization of chromatographic conditions is mainly achieved by optimizing the type of chromatographic column and the composition of the mobile phase. In order to acquire symmetrical peak shape and great separation effect in a short time, it is very important to choose a column with high separation efficiency. The chemical structures of these monomethylated ribonucleosides were illustrated in Figure 1. In our previous study, we found malic acid could enhance the detection of methylated nucleosides in HILIC-MS/MS (Guo et al., 2018a). Therefore, a hydrophilic interaction column of BEH Amide (100 mm × 2.1 mm, 1.7 μm, Waters) was selected for analysis, and malic acid was added into the mobile phase. As showed in Figure 2, it could acquire satisfactory separation for these eight ribonucleoside modifications. Besides, the analysis could be accomplished less than 6.5 min. It meant that this analytical method was quick, high throughput and fit for large clinical practice.

For the purpose of optimizing the MRM parameters, the mass spectrometer analyzed the standard solution injected by the peristaltic pump. Abundant [M + H]^+^ ions were observed in full scan ESI-MS. Then, the collision induced dissociation (CID) experiment was used to calculate the ion transitions of all analytes. In MS/MS, the ribose group can be easily eliminated due to the cleavage of the C-N bond. Taking Am and [^13^C_5_]Am as examples, abundant [M + H]^+^ ions at *m/z* 282.1 and 287.1 were observed for Am and [^13^C_5_]Am, respectively. The [M + H]^+^ ion of Am lost 146 and 151 Da was lost from [M + H]^+^ ion of [^13^C_5_]Am. Therefore, ion transition *m/z* 282.1→136.0 and *m/z* 287.1→136.0 was used for the quantification of Am and [^13^C_5_]Am, respectively. The ion transitions of other ribonucleosides and corresponding IS were shown in Supplementary Table S1. In addition, the optimized parameter values of DP, EP, CE and CXP were also listed. Under these optimized conditions, The LODs and LOQs of these monomethylated ribonucleosides can reach sub femtomole level (Supplementary Table S2).

### Validation of Analytical Method

According to the aforementioned method, the prepared calibration curve showed excellent linearities (*R* ^2^ < 0.999) in appropriate analytical ranges, and equations were showed in Table 1. The slope ratio values for these eight monomethylated ribonucleosides ranged from 94.7 to 104.7% (Table 1), which indicated the interference of matrix in this study was minimal.

**TABLE 1:** Linear equations and matrix effect values of m^6^A, Gm, m^1^G, Cm, Um and m^5^U in HILIC-MS/MS analysis.

	Linear equation	*R*^2^ value	Linear range (nM)	Matrix effect (%)
m^6^A	y = 0.3492x + 0.1053	0.9995	1–100	104.7
Gm	y = 0.1272x + 0.0308	1.0000	1–100	96.7
m^1^G	y = 0.0432x + 0.0061	1.0000	1–100	94.9
Cm	y = 0.0416x + 0.0137	0.9999	1–250	94.7
Um	y = 0.1100x + 0.0312	0.9998	1–500	99.0
m^5^U	y = 0.0173x - 0.0088	0.9999	1–500	99.4

As showed in Supplementary Table S3, the intra- and inter-day accuracy assays were in the range of 92.20–112.96% and 92.29–112.76%, respectively, and the precision of intra- and inter-day were both within 8.6%. These data indicated that sufficient reproducibility and accuracy were obtained. As showed in Supplementary Table S4, the recoveries ranged from 98.04 to 114.01% (RSD <10%), indicating an excellent recovery rate.

In a word, all these results mentioned above revealed that the established HILIC-MS/MS method could meet quantitative requirement of m^6^A, Gm, m^1^G, Cm, Um and m^5^U in human serum samples, and it was quick, accurate, sensitive, reproducible and reliable.

### Identification of Monomethylated Ribonucleoside Modifications in Human Serum

**FIGURE 3:**
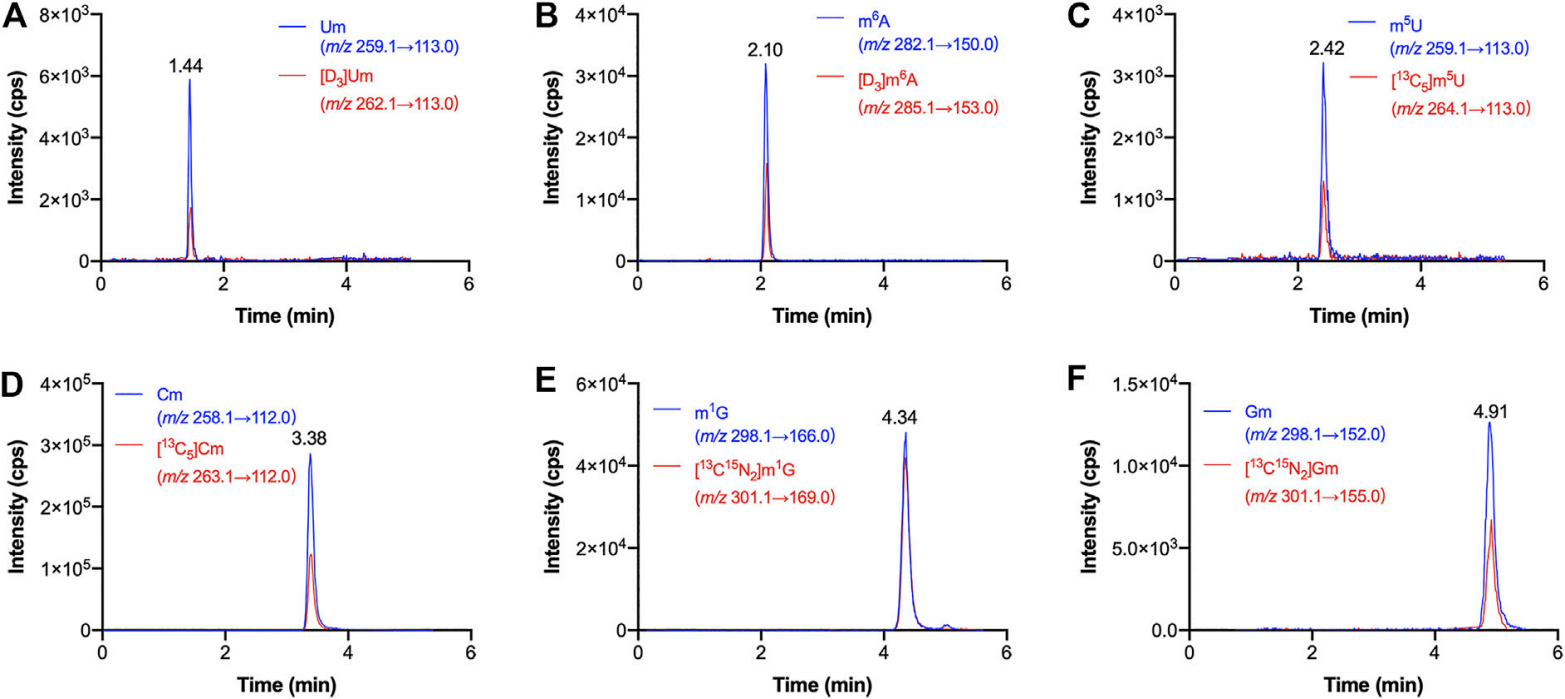
Representative MRM chromatograms of **(A)** Um, **(B)** m^6^A, **(C)** m^5^U, **(D)** Cm, **(E)** m^1^G, **(F)** Gm and spiked isotope-labeled internal standards in a serum sample.

By using this HILIC-MS/MS method, we detected these modified ribonucleosides in serum samples from 61 patients with breast cancer and 69 healthy volunteers. The results showed that m^6^A, Gm, m^1^G, Cm, Um and m^5^U were detected in all the serum samples, whereas Am and m^5^C could not be monitored due to their extremely low levels. As demonstrated in Figure 3, the retention time of Um, m^6^A, m^5^U, Cm, m^1^G and Gm were 1.44, 2.10, 2.42, 3.38, 4.34 and 4.91 min, respectively. Of note, the retention time of these compounds were consistent with their corresponding isotope-labeled internal standard. The same tandem mass spectrometry behaviors and chromatographic retention time of each modified ribonucleoside in serum as those of the isotope-labeled IS confirmed the existence of m^6^A, Gm, m^1^G, Cm, Um and m^5^U in human serum indubitably. It is worth nothing that the presence of Gm and Um in human serum was revealed for the first time, as far as we known.

### Quantification of Monomethylated Ribonucleoside Modifications in Human Serum

**FIGURE 4:**
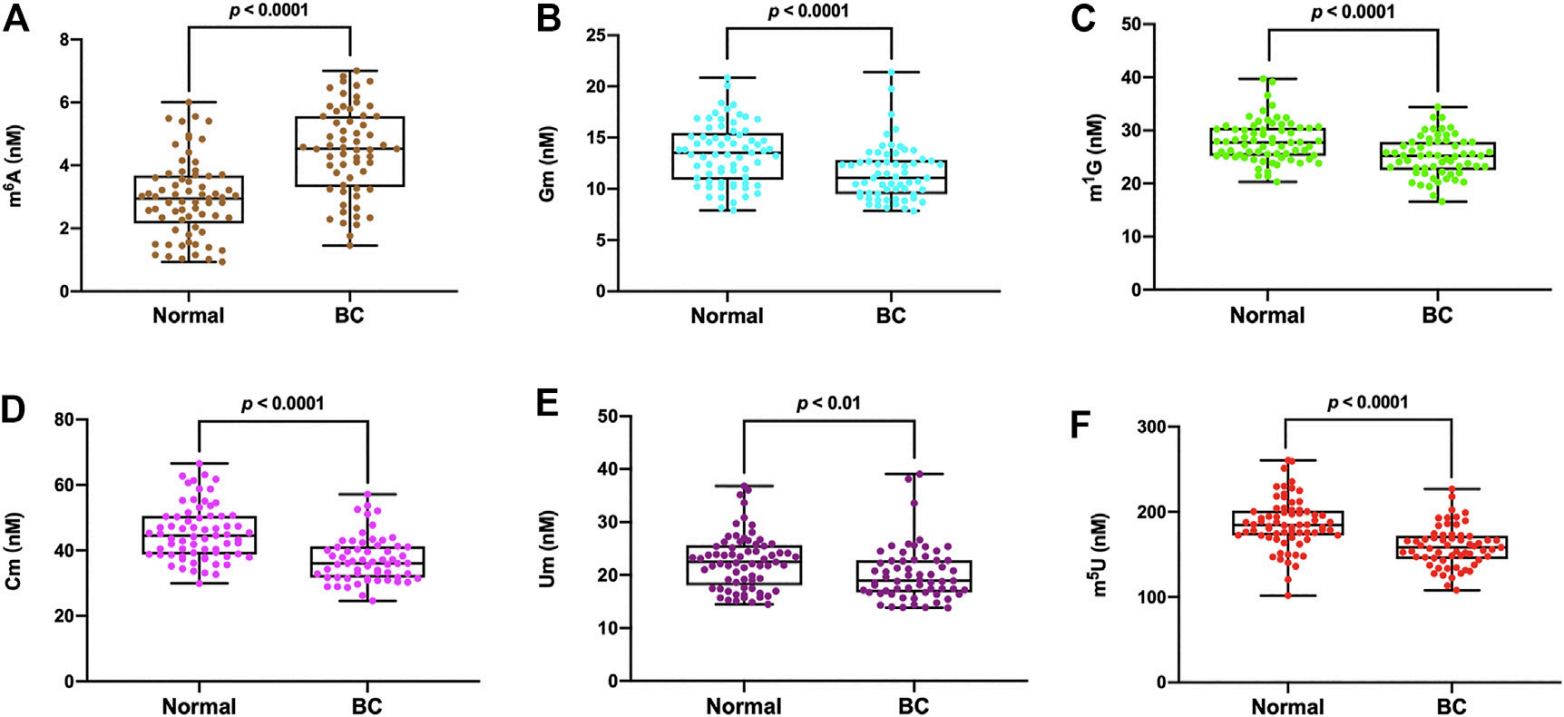
The measured concentrations of **(A)** m^6^A, **(B)** Gm, **(C)** m^1^G, **(D)** Cm, **(E)** Um, **(F)** m^5^U in serum samples and statistical analysis.

The detailed concentrations of m^6^A, Gm, m^1^G, Cm, Um and m^5^U in all the serum samples were presented in Supplementary Table S5. The measured concentrations (nM) in serum samples of m^6^A, Gm, m^1^G, Cm, Um and m^5^U from healthy volunteers were in the range of 0.93–6.01, 7.91–20.85, 20.29–39.74, 29.97–66.58, 14.47–36.83 and 101.97–260.34 nM respectively, and the average concentrations were 2.97, 13.47, 27.97, 22.60 and 186.43 nM, respectively (*n* = 69). In the serum of breast cancer patients, the concentration of m^6^A, Gm, m^1^G, Cm, Um and m^5^U were in the range of 1.45–7.01, 7.84–21.39, 16.59–34.41, 24.61–57.14, 13.83–39.07 and 108.20–226.84 nM respectively, and the average concentrations were 4.48, 11.55, 25.15, 37.31, 20.27 and 159.40 nM, respectively (*n* = 61). As illustrated in Figure 4, it was obvious that the levels of Gm, m^1^G, Cm, Um and m^5^U in serum were intensely decreased in patients with breast cancer compared to healthy controls (*p* < 0.0001 for Gm, *p* < 0.0001 for m^1^G, *p* < 0.0001 for Cm, *p* < 0.01 for Um, *p* < 0.0001 for m^5^U), but the concentration of m^6^A in breast cancer patients was much higher than that in healthy volunteers (*p* < 0.0001).

**FIGURE 5:**
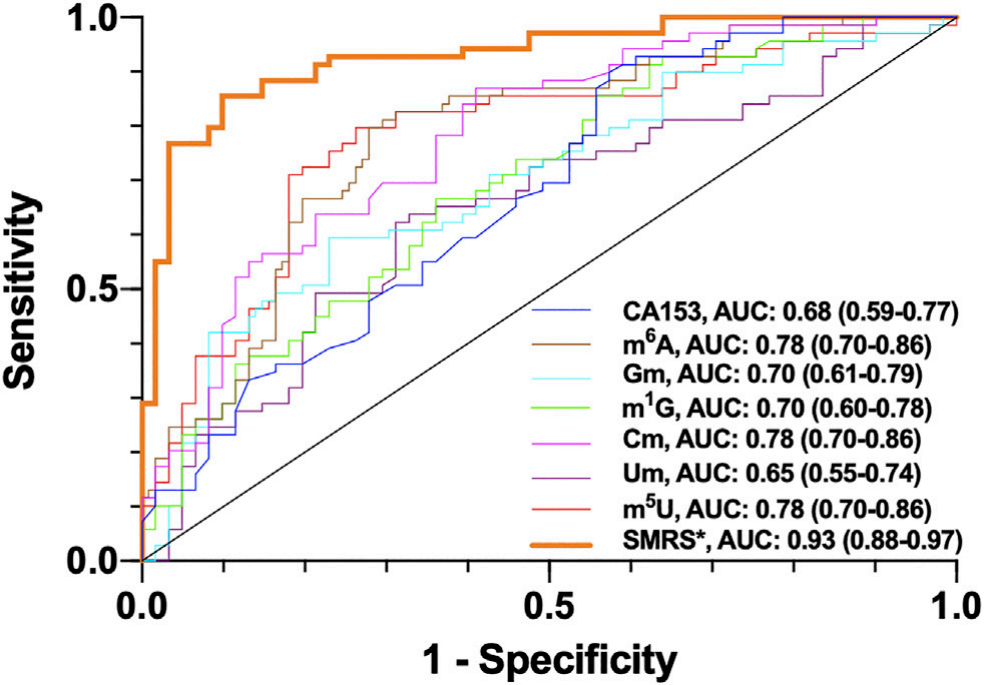
ROC analysis for m^6^A, Gm, m^1^G, Cm, Um, m^5^U, SMRS and CA153 in serum samples of breast cancer patients. *SMRS: serum monomethylated ribonucleosides score.

To verify the significance of m^6^A, Gm, m^1^G, Cm, Um and m^5^U as potential breast cancer biomarkers, receiver operating characteristic (ROC) curve was plotted. Based on the Youden index, the optimal cut-off values of m^6^A, Gm, m^1^G, Cm, Um and m^5^U were 3.76, 12.99, 25.92, 37.31,21.62 and 169.58 nM, which indicates that if the detection values break through these thresholds, it increases the risk of breast cancer. Trapezoidal rule was used to calculate area under the curve (AUC). As demonstrated in Figure 5, the AUC values were 0.78, 0.70, 0.70, 0.78, 0.65, 0.78 and 0.68 for m^6^A, Gm, m^1^G, Cm, Um, m^5^U and CA153, respectively. Most of the AUC values of ribonucleosides were higher than that of CA153, implying there might be better correlation between the levels of m^6^A, Gm, m^1^G, Cm and m^5^U in serum and the incidence of breast cancer, compared with CA153. Moreover, when all these serum monomethylated ribonucleosides were combined, the AUC could reach 0.93, which suggested the sensitivity and specificity for breast cancer diagnosis were dramatically increased. These results indicated that lower levels of Gm, m^1^G, Cm, Um and m^5^U and higher levels of m^6^A could be regarded as potential diagnostic indicators for the screening of breast cancer.

## Conclusion

In our study, a fast, robust, sensitive and trustable HILIC-MS/MS method was established for analysis of monomethylated ribonucleosides. A total of 130 serum samples from two groups containing breast cancer patients and healthy control were analyzed. Six modified ribonucleosides including m^6^A, Gm, m^1^G, Cm, Um and m^5^U were identified and quantified. Of note, to the best of our knowledge, this is the first time that Gm and Um were detected in human serum. Besides, we elucidated the differences in the contents of these monomethylated ribonucleosides between these two groups and our data, to some extent, indicated that these six modified ribonucleosides might play as potential indicators in the early detection of breast cancer.

## Data Availability

The original contributions presented in the study are included in the article/Supplementary Material, further inquiries can be directed to the corresponding author.
