# Supplementary material for: Mass Spectrometry-Based Targeted Serum Monomethylated Ribonucleosides Profiling for Early Detection of Breast Cancer
Source: Front Mol Biosci. 2021 Aug 26;8:741603. doi: 10.3389/fmolb.2021.741603 (PMC8427278; doi:10.3389/fmolb.2021.741603)
Supplement: Supplementary file 1 [file DataSheet1.PDF]

Mass spectrometry-based targeted serum monomethylated ribonucleosides profiling for early detection of breast cancer

Zhihao Fang[1], Yiqiu Hu[1], Jiani Chen[1], Kailun Xu[1], Kailai Wang[1], Shu Zheng[1,2], Cheng Guo[1,2*]

[1]Cancer Institute (Key Laboratory of Cancer Prevention and Intervention, China National Ministry of Education), The Second Affiliated Hospital, Zhejiang University School of Medicine, Hangzhou, Zhejiang 310009, China

[2]Cancer Center, Zhejiang University, Hangzhou, Zhejiang 310058, China

[*]Corresponding author:

Cheng Guo, cheng_guo@zju.edu.cn

Tel.: +86-571-87784501; Fax: +86-571-87214404.

Supplementary material

Table S1. The optimized MS conditions used for the analysis of Am, m^6A, Gm, m^1G, Cm, m^5C, Um and m^5U and isotope labelled internal standards.

Table S2. Limit of detection (LOD) and limits of quantification (LOQs) of Am, m^6A, Gm, m^1G, Cm, m^5C, Um and m^5U.

Table S3. Accuracy and precision (intra- and inter-day) for the determination of m^6A, Gm, m^1G, Cm, Um and m^5U by HILIC-MS/MS method.

Table S4. Recoveries of HILIC-MS/MS method obtained at three different spiking levels.

Table S5. General information and contents of CA153, m^6A, Gm, m^1G, Cm, Um and m^5U in serum from healthy female volunteers and breast cancer patients.

Table S1. The optimized MS conditions used for the analysis of Am, m^6A, Gm, m^1G, Cm, m^5C, Um and m^5U and isotope labelled internal standards.

Compound	MRM ion transition (*m/z*)	DP (V)	EP (V)	CE (V)	CXP (V)
Am	282.1>136.0	50	9	22	10
$[^{13}C_5]$Am	287.1>136.0	50	9	18	10
m^6A	282.1>150.0	50	12	26	10
$[D_3]m^6A$	285.1>150.0	55	7	28	12
Gm	298.1>152.0	50	7	16	12
$[^{13}C^{15}N_2]$Gm	301.1>155.0	50	7	16	12
m^1G	298.1>166.0	60	7	18	12
$[^{13}C^{15}N_2]m^1G$	301.1>169.0	55	7	20	12
Cm	258.1>112.0	45	8	16	8
$[^{13}C_5]$Cm	263.1>112.0	45	7	16	12
m^5C	258.1>126.0	50	6	16	8
$[^{13}C_5]m^5C$	263.1>126.0	50	6	16	10
Um	259.1>113.0	45	6	18	16
$[D_3]$Um	262.1>113.0	50	7	16	12
m^5U	259.1>127.0	55	9	20	14
$[^{13}C_5]m^5U$	264.1>127.0	55	7	18	12

DP declustering potential, *CE* collision energy, *EP* entrance potential, *CXP* collision cell exit potential

Table S2. Limit of detection (LOD) and limits of quantification (LOQs) of Am, m^6A, Gm, m^1G, Cm, m^5C, Um and m^5U.

	**LOD (fmol)**	**LOQ (fmol)**
Am	0.1	0.25
m^6A	0.05	0.25
Gm	0.05	0.1
m^1G	0.1	0.5
Cm	0.05	0.25
m^5C	0.05	0.5
Um	0.5	1
m^5U	1	2.5

Table S3. Accuracy and precision (intra- and inter-day) for the determination of m^{6}A, Gm, m^{1}G, Cm, Um and m^{5}U by HILIC-MS/MS method.

QC	Theoretical values (nM)	Intra-day (n = 9)			Inter-day (n = 3)		
		Mean ± SD (nM)	RSD (%)	Accuracy (%)	Mean ± SD (nM)	RSD (%)	Accuracy (%)
m^{6}A	5 (Low)	4.67 ± 0.11	2.28	93.33	4.69 ± 0.22	4.75	93.83
	10 (Medium)	9.47 ± 0.09	0.98	94.72	9.49 ± 0.19	1.98	94.92
	50 (High)	48.30 ± 1.08	2.23	96.60	48.12 ± 1.10	2.28	96.24
Gm	5 (Low)	5.09 ± 0.09	1.75	101.82	5.13 ± 0.11	2.22	102.70
	10 (Medium)	10.33 ± 0.30	2.94	103.26	10.30 ± 0.31	3.00	103.00
	50 (High)	49.78 ± 0.49	0.99	99.56	49.89 ± 0.84	1.67	99.77
m^{1}G	5 (Low)	4.61 ± 0.08	1.74	92.20	4.62 ± 0.16	3.42	92.29
	20 (Medium)	18.83 ± 0.38	2.02	94.17	19.00 ± 0.39	2.04	95.01
	100 (High)	96.14 ± 0.59	0.62	96.14	96.59 ± 1.24	1.29	96.59
Cm	5 (Low)	5.64 ± 0.09	1.57	112.96	5.64 ± 0.10	1.73	112.76
	30 (Medium)	49.85 ± 0.70	1.40	99.69	50.22 ± 0.81	1.61	100.44
	150 (High)	242.67 ± 2.34	0.97	97.07	243.72 ± 4.05	1.66	97.49
Um	5 (Low)	4.61 ± 0.27	5.91	92.29	4.65 ± 0.28	6.13	92.92
	20 (Medium)	20.59 ± 0.99	4.80	102.97	20.03 ± 1.04	5.17	100.15
	100 (High)	103.39 ± 2.76	2.67	103.39	100.93 ± 4.22	4.18	100.93
m^{5}U	20 (Low)	18.49 ± 1.33	7.22	92.45	18.74 ± 1.61	8.59	93.70
	100 (Medium)	105.29 ± 6.31	5.99	105.29	105.57 ± 3.26	3.09	105.57
	300 (High)	322.32 ± 9.66	3.00	107.44	324.91 ± 9.65	2.97	108.30

Table S4. Recoveries of HILIC-MS/MS method obtained at three different spiking levels.

	**Added amount (nM)**	**Mean ± SD (nM)**	**Average recovery (%)**	**RSD (%)**
m^6A	0	4.88±0.03	-	0.54
	2.5 (Low)	7.51±0.36	105.45	4.84
	5 (Medium)	10.79±0.13	118.20	1.19
	30 (High)	39.05±0.45	113.91	1.14
Gm	0	10.13±0.07	-	0.71
	2.5 (Low)	12.68±0.55	102.12	4.35
	10 (Medium)	20.30±0.99	101.78	4.88
	50 (High)	59.70±0.11	99.15	0.18
m^1G	0	21.96±0.30	-	1.37
	5 (Low)	27.00±0.38	100.96	1.39
	20 (Medium)	42.00±0.32	100.24	0.77
	60 (High)	81.92±1.54	99.94	1.88
Cm	0	39.26±0.28	-	0.71
	6 (Low)	45.76±1.35	108.29	2.95
	30 (Medium)	72.82±0.29	111.88	0.40
	150 (High)	199.19±2.05	106.62	1.03
Um	0	18.57±1.74	-	9.35
	5 (Low)	24.23±1.07	113.20	4.40
	20 (Medium)	38.18±1.41	98.04	3.68
	60 (High)	77.46±5.75	98.15	7.42
m^5U	0	122.59±11.84	-	9.66
	50 (Low)	179.60±13.36	114.01	7.44
	150 (Medium)	282.37±18.33	106.52	6.49
	300 (High)	430.01±11.68	102.47	2.72

Table S5. General information and contents of CA153, m^{6}A, Gm, m^{1}G, Cm, Um and m^{5}U in serum from healthy female volunteers and breast cancer patients.

NO.	Age (years)	CA153 (U/mL)	m^{6}A (nM)	Gm (nM)	m^{1}G (nM)	Cm (nM)	Um (nM)	m^{5}U (nM)
N1	29	4.0	2.61±0.21	14.30±0.41	25.40±0.64	61.77±0.59	22.46±0.05	259.35±51.8
N2	51	5.6	3.10±0.18	16.88±0.05	25.96±0.15	55.55±0.65	29.72±1.50	140.35±20.8
N3	55	8.1	2.38±0.01	14.71±0.26	23.85±0.42	45.09±0.09	24.49±1.67	175.85±0.2
N4	39	8.1	1.80±0.21	14.98±0.49	31.72±0.09	50.59±0.70	23.49±0.67	177.06±15.7
N5	39	15.0	3.37±0.18	13.79±0.27	31.25±1.29	35.78±0.76	25.41±0.53	197.43±4.1
N6	42	5.8	3.22±0.07	12.38±0.04	30.87±0.48	38.19±0.08	23.29±1.32	204.19±16.7
N7	51	14.3	3.02±0.31	18.38±0.84	29.58±0.04	43.64±0.07	25.15±0.21	187.59±0.8
N8	57	11.9	2.40±0.12	13.99±0.31	27.85±0.63	42.87±0.58	23.74±0.58	199.37±16.7
N9	35	10.1	1.95±0.07	15.11±0.52	31.14±0.14	51.57±0.43	23.79±1.08	235.26±3.9
N10	73	11.8	2.82±0.05	14.56±0.21	30.07±0.28	50.28±0.59	28.07±0.28	260.34±4.5
N11	63	7.7	2.85±0.21	16.91±0.03	39.15±1.05	42.07±0.39	25.79±5.31	230.08±5.6
N12	71	5.4	3.56±0.01	11.46±0.47	27.75±0.16	42.18±0.94	19.84±2.87	211.09±20.8
N13	46	5.4	1.40±0.05	14.97±0.04	27.96±0.24	54.58±0.16	26.31±1.16	187.77±4.8
N14	67	7.7	4.11±0.32	16.50±0.10	30.24±0.01	50.40±0.27	27.31±0.21	194.15±7.5
N15	65	3.6	2.27±0.14	17.42±0.87	28.67±0.48	61.37±0.62	21.73±0.17	181.93±4.1
N16	67	3.4	2.82±0.06	18.21±0.82	22.68±0.25	60.70±0.50	36.05±1.91	198.25±7.3
N17	71	6.1	3.01±0.11	17.79±0.49	32.66±0.32	53.17±0.24	26.22±4.76	178.58±18.4
N18	65	8.4	2.55±0.06	13.71±0.45	24.62±0.46	40.37±0.39	22.29±2.15	180.70±24.4
N19	47	4.6	2.65±0.05	13.89±0.28	32.50±0.91	43.60±0.22	26.97±0.54	172.27±12.1
N20	57	5.6	1.49±0.05	8.14±0.05	23.85±0.61	37.40±0.19	15.10±1.06	120.51±1.8

N21	72	5.5	1.88±0.00	9.21±0.23	21.40±0.23	29.97±0.20	14.87±0.37	101.97±14.9
N22	50	5.7	2.35±0.05	9.15±0.05	20.29±0.29	32.73±0.58	17.58±3.25	147.14±5.4
N23	53	5.1	2.83±0.03	14.42±1.29	24.87±0.31	45.46±0.75	25.47±1.26	178.14±1.2
N24	47	6.7	3.84±0.03	16.14±0.11	36.61±0.77	46.37±0.03	27.46±1.41	167.75±4.1
N25	49	5.6	1.45±0.05	13.35±0.68	28.09±0.29	38.83±0.60	22.16±1.73	149.94±4.4
N26	40	4.1	3.67±0.04	10.24±0.39	25.04±0.08	33.71±1.23	17.47±0.75	172.57±13.8
N27	52	10.9	2.05±0.07	14.72±0.55	30.16±0.06	44.54±0.26	21.39±0.47	200.46±4.8
N28	47	8.1	2.82±0.05	15.67±0.05	27.02±0.79	46.69±0.11	24.18±0.64	176.48±28.0
N29	53	10.3	3.04±0.09	10.46±0.11	24.15±0.41	34.43±0.01	16.95±0.46	224.86±14.3
N30	42	4.6	2.57±0.16	11.14±0.85	21.48±0.09	38.70±0.02	23.73±2.25	184.60±17.6
N31	62	11.7	3.08±0.07	15.28±0.41	28.25±0.05	46.72±0.30	23.89±1.73	195.99±7.4
N32	53	6.2	3.61±0.03	10.14±0.44	30.45±0.03	37.53±0.49	16.58±0.65	149.45±17.4
N33	62	7.5	2.45±0.05	10.32±0.03	27.72±0.02	40.47±0.28	15.74±0.48	163.67±1.4
N34	52	16.8	2.59±0.15	15.10±0.16	28.52±1.51	47.02±0.34	21.86±1.07	189.75±1.4
N35	44	18.6	2.98±0.01	10.65±0.98	32.38±0.08	35.26±0.14	16.98±1.07	229.53±8.4
N36	43	5.0	2.34±0.02	13.82±0.16	27.00±0.01	50.00±0.98	23.02±3.35	148.03±0.2
N37	39	9.5	3.31±0.02	20.07±0.01	25.97±0.30	66.58±1.41	35.15±0.77	178.60±0.2
N38	47	6.3	3.22±0.03	13.11±0.09	26.03±0.01	44.89±0.24	21.76±0.38	175.72±13.0
N39	41	5.1	6.01±0.22	10.74±0.28	25.61±0.24	40.37±0.29	16.23±2.13	169.82±3.0
N40	50	7.7	5.50±0.00	16.81±0.08	25.22±0.27	55.08±1.42	30.87±2.51	151.37±21.5
N41	55	10.3	4.67±0.15	17.27±0.66	25.19±0.54	55.28±0.38	29.44±0.30	194.73±10.7
N42	43	8.2	1.16±0.04	11.93±0.31	22.20±0.12	47.43±0.17	16.02±0.80	192.74±15.7
N43	46	6.6	3.80±0.02	14.32±0.30	39.74±0.58	50.68±0.46	26.69±3.16	221.25±8.5
N44	42	7.5	4.16±0.17	13.08±0.45	26.27±0.35	50.51±0.43	21.01±0.95	217.45±3.6
N45	50	6.3	1.10±0.04	10.11±0.05	25.32±0.59	39.28±0.44	16.32±0.15	162.20±12.6
N46	45	6.0	1.56±0.09	11.68±0.41	26.23±0.43	33.18±0.55	19.37±0.49	188.79±10.2

N47	48	4.2	1.03±0.17	17.05±1.28	28.04±0.78	58.75±0.39	33.64±3.16	144.35±6.4
N48	41	18.6	4.96±0.05	16.80±0.33	24.44±0.33	63.18±0.53	24.43±0.35	167.59±11.4
N49	60	5.0	1.49±0.02	9.53±0.42	25.59±0.17	37.89±0.34	14.47±0.45	196.37±5.0
N50	42	11.6	3.70±0.09	8.66±0.55	30.53±0.30	38.73±0.19	15.82±1.93	174.42±10.1
N51	57	3.5	5.40±0.08	11.05±0.17	29.97±0.39	35.62±0.21	17.41±0.36	227.91±7.1
N52	47	12.6	3.49±0.12	15.63±0.39	34.73±1.79	58.82±0.24	26.54±1.02	251.25±26.4
N53	41	14.8	1.48±0.08	12.25±0.34	29.42±0.18	42.32±0.36	19.01±1.41	184.86±2.1
N54	44	6.0	4.87±0.09	10.18±0.11	26.18±0.08	38.98±0.26	18.58±0.01	175.67±17.4
N55	47	3.7	3.18±0.09	13.34±0.11	31.93±0.18	43.30±0.74	24.13±1.31	200.41±13.3
N56	51	10.8	1.16±0.01	12.35±0.23	33.74±0.38	48.98±0.41	21.77±0.63	218.50±6.0
N57	52	8.1	3.10±0.05	20.85±0.03	30.89±0.10	62.80±0.91	36.83±0.98	136.17±7.5
N58	39	8.3	3.74±0.00	10.55±0.12	24.87±0.15	36.15±0.30	17.48±1.81	187.78±24.2
N59	45	4.0	2.95±0.07	7.91±0.27	24.48±0.49	39.43±0.58	15.69±1.72	185.14±27.8
N60	44	5.6	1.02±0.04	14.40±0.19	29.27±1.11	47.44±1.14	23.11±0.60	170.14±0.4
N61	48	6.3	3.07±0.04	12.59±0.01	26.92±0.12	44.24±0.72	22.13±1.60	183.01±9.7
N62	50	5.1	0.93±0.00	13.22±0.07	23.80±0.07	45.33±0.31	20.55±0.45	202.12±35.6
N63	47	10.9	2.83±0.03	15.97±0.05	25.51±0.05	53.67±0.24	25.90±0.08	204.50±7.1
N64	40	7.3	4.42±0.05	11.80±0.34	30.20±0.05	47.43±0.53	21.76±2.40	202.18±8.1
N65	48	10.4	1.30±0.01	13.32±0.15	31.34±0.01	47.01±0.08	23.85±1.40	181.75±22.2
N66	45	3.9	3.70±0.00	9.21±0.39	27.29±0.01	37.98±0.07	15.28±0.41	184.05±1.3
N67	50	10.9	5.55±0.06	10.25±0.04	23.52±0.24	40.50±0.35	19.17±1.05	172.54±22.1
N68	50	10.6	4.83±0.10	12.00±0.34	30.69±0.14	41.91±0.89	19.10±0.31	212.23±18.8
N69	45	6.3	5.41±0.08	13.53±0.43	27.76±0.07	38.19±1.11	23.46±0.10	175.53±4.6
BC1	74	8.6	4.03±0.14	8.47±0.09	25.10±0.09	28.69±0.25	14.30±1.39	226.84±1.2
BC2	55	18.3	3.24±0.18	12.36±0.38	23.06±0.11	36.73±0.06	20.06±0.42	133.99±10.4
BC3	58	27.7	4.65±0.52	13.76±0.06	29.14±0.45	43.40±0.17	25.38±3.76	160.89±3.5

BC4	66	7.0	4.53±0.12	8.91±0.17	27.12±0.15	31.77±0.20	17.39±1.62	137.83±3.3
BC5	53	5.0	4.00±0.03	9.45±0.09	25.83±0.02	31.78±0.61	15.57±0.36	193.06±1.9
BC6	48	6.2	2.64±0.00	12.42±0.13	23.18±0.25	39.73±0.05	22.77±1.24	146.64±0.2
BC7	31	5.9	4.53±0.44	13.40±0.42	25.60±0.09	42.97±0.69	25.63±3.24	172.91±2.2
BC8	38	7.6	3.61±0.14	10.39±0.08	24.66±0.18	30.46±0.16	20.60±0.27	134.51±7.1
BC9	41	14.5	3.81±0.55	15.82±0.17	25.48±1.44	41.04±1.30	24.29±3.70	150.97±16.2
BC10	44	574.5	6.68±0.16	8.98±0.09	32.44±0.19	35.78±0.86	16.74±0.22	122.65±9.0
BC11	57	14.9	6.29±0.18	10.41±0.34	26.12±0.00	37.09±0.37	17.38±0.90	152.33±7.5
BC12	67	5.9	4.49±0.10	7.84±0.36	22.20±0.14	29.67±0.02	13.91±0.07	145.95±14.3
BC13	69	5.3	6.54±0.03	8.69±0.13	24.09±0.05	30.91±0.28	13.97±1.46	166.90±15.5
BC14	69	8.3	5.02±0.20	12.61±0.01	25.23±0.45	42.44±0.26	21.29±3.70	159.89±4.5
BC15	38	4.4	5.10±0.10	8.84±0.17	20.93±0.70	26.20±0.18	15.44±0.38	127.76±14.5
BC16	61	4.3	5.72±0.00	11.08±0.52	20.90±0.05	41.55±0.25	16.50±0.21	166.52±10.7
BC17	64	31.8	6.68±0.07	9.96±0.10	22.02±0.40	36.05±0.72	17.44±0.59	154.76±0.8
BC18	45	5.7	5.89±0.50	9.95±0.05	25.29±0.21	34.13±0.52	16.38±0.59	108.20±3.2
BC19	40	6.1	6.01±0.31	9.04±0.08	30.33±0.52	38.37±0.43	18.95±0.62	193.90±3.8
BC20	52	26.0	5.56±0.47	8.08±0.03	25.22±0.23	33.32±0.14	16.39±1.19	143.18±5.1
BC21	64	10.7	4.91±0.08	9.62±0.32	24.33±0.30	30.96±0.06	17.40±1.11	161.80±7.9
BC22	49	13.0	5.58±0.42	12.33±0.11	23.19±0.12	37.80±0.36	20.11±1.63	173.30±15.8
BC23	62	8.3	5.36±0.19	9.56±0.42	24.22±0.00	31.35±0.47	18.13±1.78	199.06±2.3
BC24	59	5.6	5.88±0.02	10.75±0.31	25.76±0.25	40.07±0.89	17.02±1.10	185.14±5.0
BC25	36	11.8	5.41±0.08	13.82±0.17	23.24±0.66	40.37±0.35	25.86±0.31	169.34±2.9
BC26	47	7.70	4.25±0.04	11.47±0.63	25.89±0.61	35.28±0.23	19.94±0.56	186.16±2.7
BC27	50	23.80	4.11±0.31	8.50±0.10	24.05±0.47	31.51±0.94	16.75±0.26	189.52±12.2
BC28	70	8.10	3.37±0.20	11.58±0.57	25.25±0.22	34.04±0.44	22.87±2.74	166.89±10.7
BC29	57	15.60	3.17±0.05	9.93±0.68	21.90±0.13	36.11±0.24	14.38±0.09	148.86±4.4

BC30	54	14.60	5.88±0.53	13.60±0.18	32.46±0.05	45.46±1.06	21.51±1.63	157.11±10.0
BC31	38	14.70	4.82±0.27	12.38±0.07	30.55±0.51	43.02±0.19	21.14±1.11	131.71±6.6
BC32	80	14.60	6.84±0.28	9.52±0.15	28.41±0.02	31.52±0.06	18.85±1.31	165.48±2.4
BC33	60	12.60	6.19±0.76	10.63±0.24	25.55±0.14	34.29±0.47	19.07±1.00	202.33±33.7
BC34	29	10.50	4.37±0.19	10.01±0.39	20.22±0.28	30.33±0.21	17.19±1.29	167.90±14.3
BC35	41	27.40	6.47±0.14	13.54±0.60	29.04±0.03	43.16±1.14	23.54±0.70	217.80±29.9
BC36	59	18.1	3.02±0.05	11.48±0.13	22.93±0.31	35.78±0.17	22.69±0.48	143.64±3.8
BC37	50	5.2	3.25±0.04	12.73±0.29	23.79±0.06	43.93±0.74	22.29±0.26	152.66±6.8
BC38	40	8.1	4.50±0.13	14.15±0.17	22.98±0.02	41.13±0.13	26.65±1.41	127.73±3.3
BC39	67	6.1	7.01±0.08	8.37±0.47	26.79±0.64	28.95±0.33	14.76±1.06	183.95±7.5
BC40	50	5.7	2.29±0.06	10.61±0.06	20.63±0.37	30.86±0.27	18.09±0.99	147.85±24.6
BC41	56	96.2	2.33±0.15	12.40±0.06	22.07±0.10	31.77±0.47	20.75±1.86	147.05±6.4
BC42	70	17.1	4.64±0.19	9.24±0.11	27.09±0.36	32.87±0.07	15.71±1.11	165.83±8.8
BC43	38	4.0	4.58±0.22	11.18±0.49	34.40±1.02	38.35±0.59	18.59±1.36	153.28±0.5
BC44	44	4.4	2.52±0.16	21.39±0.28	30.18±0.32	57.14±0.82	39.07±2.88	163.03±9.6
BC45	42	28.5	2.12±0.13	8.91±0.39	17.73±0.20	28.98±0.12	16.34±0.32	137.30±1.5
BC46	56	27.7	4.75±0.09	17.25±0.32	28.13±0.28	52.09±0.54	33.56±3.86	174.47±5.7
BC47	67	9.2	1.76±0.12	8.16±0.15	16.59±0.54	24.61±0.05	13.88±1.45	113.44±10.7
BC48	52	6.0	4.09±0.12	12.49±0.50	29.38±0.33	32.86±0.26	19.64±0.67	125.52±12.9
BC49	46	6.6	3.27±0.22	12.68±0.42	19.69±0.03	41.30±0.05	17.13±0.27	166.75±22.3
BC50	36	6.7	3.77±0.12	9.09±0.15	19.43±0.28	36.56±0.25	13.83±1.03	133.57±20.6
BC51	48	7.7	4.97±0.09	10.46±0.01	30.30±0.28	37.22±0.12	18.79±0.86	158.34±15.1
BC52	48	23.6	4.57±0.03	19.77±0.15	29.21±0.37	47.76±0.36	38.15±1.78	160.57±9.1
BC53	46	6.0	4.29±0.04	13.49±0.06	20.25±0.07	39.68±0.21	25.37±1.49	171.44±12.9
BC54	43	8.1	1.45±0.03	12.59±0.32	20.17±0.04	39.63±0.16	20.83±3.04	152.17±5.3
BC55	55	7.9	5.56±0.08	10.51±0.45	27.82±0.23	33.23±0.75	18.57±1.30	155.99±11.9

BC56	63	26.2	2.74±0.04	11.05±0.25	27.69±0.10	33.00±0.29	17.55±0.03	168.79±11.2
BC57	42	7.2	2.37±0.09	14.27±0.05	21.72±0.14	51.11±0.09	25.53±3.12	192.87±24.5
BC58	69	25.0	5.28±0.23	14.23±0.16	27.80±0.38	52.59±0.08	22.67±1.00	172.34±10.5
BC59	50	17.7	4.28±0.05	15.34±0.46	27.81±0.27	53.68±0.70	24.51±1.00	130.30±2.9
BC60	50	14.7	5.80±0.10	12.90±0.22	28.02±0.08	37.18±0.72	22.78±1.82	153.84±0.6
BC61	29	20.9	2.17±0.09	12.20±0.05	23.26±0.25	36.10±0.21	24.55±0.47	148.60±3.3
